# The Synergistic Effect of Adsorption-Photocatalysis for Removal of Organic Pollutants on Mesoporous Cu_2_V_2_O_7_/Cu_3_V_2_O_8_/g-C_3_N_4_ Heterojunction

**DOI:** 10.3390/ijms232214264

**Published:** 2022-11-17

**Authors:** Jian Feng, Xia Ran, Li Wang, Bo Xiao, Li Lei, Jinming Zhu, Zuoji Liu, Xiaolan Xi, Guangwei Feng, Zeqin Dai, Rong Li

**Affiliations:** Engineering Research Center for Molecular Medicine, School of Basic Medical Sciences, Guizhou Medical University, Guiyang 550025, China

**Keywords:** Cu_2_V_2_O_7_/Cu_3_V_2_O_8_/g-C_3_N_4_ heterojunctions, adsorption, photocatalysis, degradation, Z-scheme

## Abstract

Cu_2_V_2_O_7_/Cu_3_V_2_O_8_/g-C_3_N_4_ heterojunctions (CVCs) were prepared successfully by the reheating synthesis method. The thermal etching process increased the specific surface area. The formation of heterojunctions enhanced the visible light absorption and improved the separation efficiency of photoinduced charge carriers. Therefore, CVCs exhibited superior adsorption capacity and photocatalytic performance in comparison with pristine g-C_3_N_4_ (CN). CVC-2 (containing 2 wt% of Cu_2_V_2_O_7_/Cu_3_V_2_O_8_) possessed the best synergistic removal efficiency for removal of dyes and antibiotics, in which 96.2% of methylene blue (MB), 97.3% of rhodamine B (RhB), 83.0% of ciprofloxacin (CIP), 86.0% of tetracycline (TC) and 80.5% of oxytetracycline (OTC) were eliminated by the adsorption and photocatalysis synergistic effect under visible light irradiation. The pseudo first order rate constants of MB and RhB photocatalytic degradation on CVC-2 were 3 times and 10 times that of pristine CN. For photocatalytic degradation of CIP, TC and OTC, it was 3.6, 1.8 and 6.1 times that of CN. DRS, XPS VB and ESR results suggested that CVCs had the characteristics of a Z-scheme photocatalytic system. This study provides a reliable reference for the treatment of real wastewater by the adsorption and photocatalysis synergistic process.

## 1. Introduction

In recent decades, with the rapid development of industrialization, environmental pollution has become increasingly serious. More and more organic chemicals have been released into the environment. Water pollution has become one of the major obstacles to the sustainable development of human society [1]. Various organic pollutants, such as dyes and antibiotics, are seriously harmful to the ecological environment and human health [2]. Many traditional techniques have been developed to remove the organic pollutants in wastewater, including the bioelectrochemical method [3], electrochemical advanced oxidation processes [4], flocculent precipitation [5], physisorption [6], biological degradation, incineration, membrane filtration, etc. [7]. However, these traditional techniques suffer from the drawbacks, such as not being suitable for low concentrations of pollutant, high operation costs, low removal efficiency and secondary pollution. To date, the integration of adsorption and photocatalysis has been regarded as the most promising technology for the elimination of low-concentration contaminants [8,9,10]. This technology can combine the advantages of adsorption and photocatalysis, such as high efficiency, low cost, wide availability to adsorbates, superior recoverability and less secondary pollution. The adsorption process can ameliorate the accumulation of contaminants on the catalyst surface from the wastewater and conduce to improving the photocatalytic degradation efficiency. The photocatalysis can ultimately mineralize the contaminants to H_2_O and CO_2_ under light irradiation at room temperature, which reproduces the surface of the catalyst for the next adsorption process [11,12,13]. Therefore, the development of semiconductor materials with superior adsorption and photocatalysis performance in wastewater treatment has extensively aroused research interests [14,15,16].

At present, MXenes [17], hydrogels [18,19], graphene-like nanomaterials [20] and their composites have been extensively studied in the photocatalysis and adsorption fields. Among them, graphitic carbon nitride (CN) is considered as one of the most promising 2D materials for environmental remediation based on its impressive merits of low cost, high stability, proper band gap for visible light harvesting and low toxicity [21,22]. Many researchers have reported the adsorption capability of CN for wastewater treatment [16,23,24]. However, the adsorption and photocatalysis performance of pristine CN still sustains small specific surface area, fast recombination of photoinduced charge carriers and narrow visible light absorption [25]. Accordingly, lots of technologies such as elemental doping [25], heterostructure construction [26,27,28], morphology control [29,30] and defect engineering [31] have been devoted to ameliorating its adsorption and photocatalysis performance. Wherein, controlling the morphology of CN and constructing CN-based heterostructures are especially regarded as effective strategies to extend the visible light absorption range, increase the specific surface area and adsorption sites and accelerate the separation of photoinduced charge carriers [32]. Ultrathin CN nanosheets acquired by the exfoliation process have a much larger specific surface area than bulk CN, which is instrumental in promoting the adsorption capability [30]. Moreover, the diffusion distance of photoinduced charge carriers is shortened in ultrathin CN nanosheets. It can reduce the recombination of photoinduced charge carriers and boost the photocatalytic performance [33]. Additionally, the construction of CN-based heterojunctions via loaded metal oxides is another method to improve the photocatalytic performance [32]. The spatial separation of photoinduced charge carriers at the interface of two semiconductors will prohibit their recombination. Moreover, the formation of CN-based heterojunctions can promote band matching and light absorption, and enhance photocatalytic reaction activity [21]. Therefore, manufacturing CN-based heterojunctions with ultrathin structures should be a feasible strategy to raise the adsorption and photocatalysis synergy for removal of contaminants.

Copper vanadates are a class of catalysts with band gap of ~2 eV, making them suitable for visible light absorption [34]. They thus exhibit dye degradation activity as photocatalysts [35,36] and water splitting property as photoanode candidates [37,38,39]. High-throughput research results show that copper vanadates should be a novel class of materials for photocatalytic application [38,40]. Copper vanadates with different stoichiometric ratios, such as CuV_2_O_6_ [37], Cu_2_V_2_O_7_ [34,36] and Cu_3_V_2_O_8_ [34,41], can be prepared by changing the molar ratio of Cu:V and the synthesis method. Khan et al. synthesized CuV_2_O_6_ and Cu_2_V_2_O_7_ via a sonication assisted sol–gel method with a band gap of 1.84 eV and 2.2 eV, respectively [35]. CuV_2_O_6_, Cu_2_V_2_O_7_ and Cu_5_V_2_O_10_ were prepared using a sol–gel method by adjusting the Cu:V [39]. Then, these copper vanadates were applied as photocatalysts on the selective oxidation of cyclohexane. Keerthana et al. synthesized β-Cu_2_V_2_O_7_, CTAB-β-Cu_2_V_2_O_7_ and PVP-Cu_3_V_2_O_8_ by a hydrothermal method [36]. The bandgaps were 3.09 eV, 2.97 eV and 2.28 eV, respectively. PVP-Cu_3_V_2_O_8_ presented 96%, 77% and 96% photocatalytic removal efficiency for MB, RhB and malachite green dyes. β-Cu_2_V_2_O_7_ nanorods were also synthesized and the photocatalytic degradation property was assessed, with 81.85% of MB degraded within 60 min of visible light irradiation [42]. Cu_3_V_2_O_8_ nanoparticles were produced via a precipitation approach using Schiff base as the ligand. They exhibited 79% MB degradation efficiency under UV irradiation [43]. Actually, despite the favorable band gaps for visible light harvesting, copper vanadates still manifested low photocatalytic efficiencies [37].

To date, element doping and heterojunction constructing are the major approaches that have been investigated to enhance photocatalytic efficiency of copper vanadates. Indium-doped CuV_2_O_6_wasprepared by the hydrothermal method and it revealed efficient optical absorption from the UV to visible region with gap energy of 1.96 eV [44]. About 95% of RhB was removed during visible light irradiation for 120 min over CuV_2_O_6_:In^3+^. Contrastively, only 57% of RhB was eliminated over CuV_2_O_6_ under the same conditions. Furthermore, copper vanadate-based heterojunctions, involving in Cu_2_V_2_O_7_/g-C_3_N_4_ [45], Cu_2_V_2_O_7_/CoFe_2_O_4_/g-C_3_N_4_ [46], Cu_2_O/Cu_2_V_2_O_7_ [47], r-GO/β-Cu_2_V_2_O_7_/TiO_2_ [48], β-Cu_2_V_2_O_7_/Zn_2_V_2_O_6_ [49] and Cu_2_V_2_O_7_/Cu_3_V_2_O_8_ [41] were manufactured to enhance photocatalytic activities. Although the relevant studies have shown important progress, more extensive efforts should be made to enhance photocatalytic efficiency of copper vanadates. Herein, Cu_2_V_2_O_7_/Cu_3_V_2_O_8_ (CV) was loaded on ultrathin CN nanosheets to form CVC heterojunctions. The exfoliation of bulk CN increased the specific surface area and shortened the diffusion distance of photoinduced charge carriers. The construction of CVCs further accelerated the separation of charge carriers. Thus, the adsorption capability and photocatalytic degradation activity could be remarkably enhanced. The removal of CIP, TC, OTC, MB and RhB was investigated to discuss the synergistic effect of adsorption and photocatalysis.

## 2. Results and Discussion

**Figure 1:**
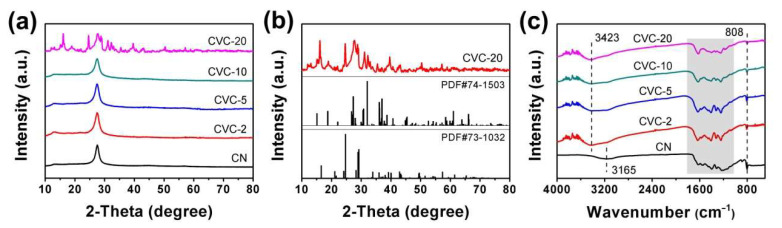
(**a**) XRD patterns of CN, CVC-2, CVC-5, CVC-10 and CVC-20, (**b**) XRD patterns of CVC-20, monoclinic Cu_3_V_2_O_8_ (PDF#74-1503) and monoclinic Cu_2_V_2_O_7_ (PDF#73-1032), (**c**) FTIR spectra of CN, CVC-2, CVC-5, CVC-10 and CVC-20.

The XRD patterns of CN, CVC-2, CVC-5, CVC-10 and CVC-20 heterojunctions are depicted in Figure 1a. All samples presented two peaks at 13.0° and 27.6°, corresponding to (110) and (022) crystal planes of CN. They demonstrated the existence of CN in these samples [16]. The diffraction peaks of CV were undetectable in CVC-2, CVC-5 and CVC-10. This may have resulted from the low dosage of CV in these heterojunctions, which could not cause the change of the chemical skeleton and structure of CN. This result was previously clarified by the literature [50,51]. Moreover, the diffraction peak intensity of CV in CVC-2, CVC-5 and CVC-10 were weaker while CV nanoparticles were covered by CN nanosheets [51]. Comparatively, the diffraction peaks of both CN and CV were all found in CVC-20. In fact, the existence of CV in CVC-2, CVC-5 and CVC-10 could be corroborated by EDS and elemental mapping results. SEM images and corresponding EDS of CVC-2 are displayed in Figure S1. It was revealed that the mass percentage of CV in CVC-2 was about 2.11%, which was well in accordance with the value calculated from the content of Cu(NO_3_)_3_ and NH_4_VO_3_ in the precursor. The chemical composition and uniformity of CVC-2 were confirmed by the elemental mappings of C, N, O, Cu and V. All elements can be observed in Figure S2b–f. The uniformity of C, N, O, Cu and V demonstrated the homogeneous distribution of CV on CN nanosheets. In addition, the XRD patterns of CV conformed well to the monoclinic phase Cu_2_V_2_O_7_ (PDF#73-1032) and monoclinic Cu_3_V_2_O_8_ (PDF#74-1503), indicating that CV was the composite of Cu_2_V_2_O_7_ and Cu_3_V_2_O_8_ (Figure 1b). These XRD results definitely proved the successful formation of CVCs.

The FTIR spectra of CN, CVC-2, CVC-5, CVC-10 and CVC-20 are shown in Figure 1c. The sharp adsorption peaks at 806 and 886 cm^−1^ were assigned to breathing vibration and N-H deformation vibration mode of CN s-triazine, respectively. The characteristic adsorption band in the range of 1200–1700 cm^−1^ was ascribed to the stretching vibrations of C–N and C=N of aromatic CN heterocycles [52]. The broad adsorption band at 2900–3500 cm^−1^ was attributed to N-H stretching vibration of residual unpolymerized amino groups. This adsorption band of CN centered at 3165 cm^−1^ shifted to 3423 cm^−1^ while CV was loaded on the CN nanosheets. This change of the peak position implied the strong interaction between CV nanoparticles and CN nanosheets [32].

**Figure 2:**
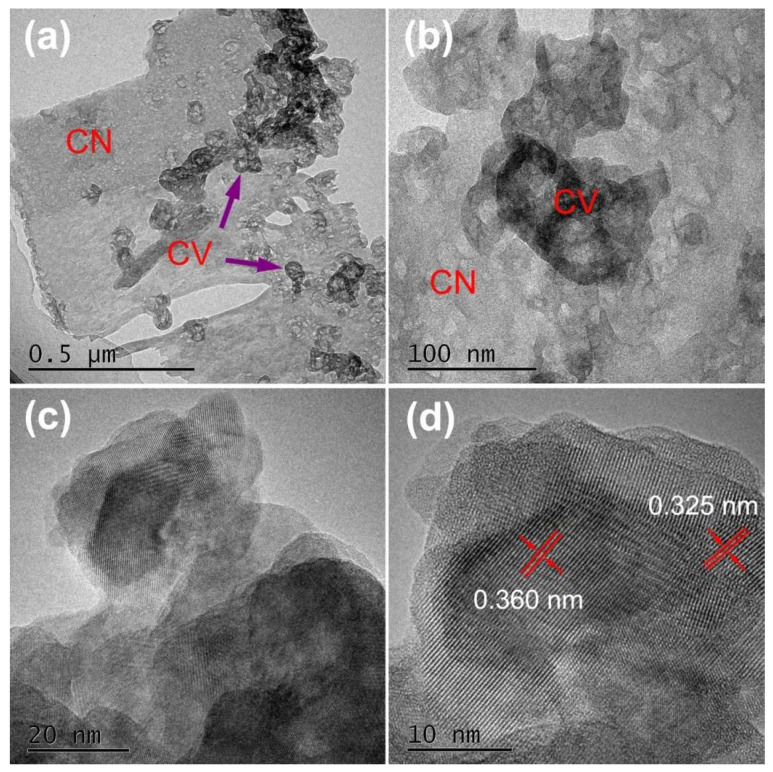
(**a**,**b**) TEM and (**c**,**d**) high-resolution TEM images of CVC-2, the scale bar represented 0.5 μm, 100 nm, 20 nm and 10 nm, respectively.

TEM and HRTEM images of CVC-2 are displayed in Figure 2. As shown in Figure 2a,b, there was an ultrathin layered structure of CN nanosheets. It profited from the thermal etching effect in the reheating synthesis process. Especially, the porous structure of CVC-2 could also be observed (Figure 2a,b). The pore diameter was between several and tens of nanometers [50]. This porous structure might originate from the generation of NH_3_ and HCl in the polymerization process of dicyandiamine and NH_4_Cl [53]. The ultrathin and porous structure increased the specific surface area of CVC-2, which was proved by BET results. The increased specific surface area could improve the adsorption capability of CVC-2. CV nanoparticles with evident aggregation were also observed in Figure 2a,b. Therefore, the diameters of CV nanoparticles could not be measured accurately from the TEM image. They were estimated to be 50–100 nm in diameter. From Figure 2a,b, it can also be seen that CV nanoparticles were located on CN nanosheets. The spacing of lattice fringes of 0.360 and 0.325 nm are distinctly observed in HRTEM images illustrated in Figure 2c,d. This was consistent with the (200) and (111) crystal planes of monoclinic phase Cu_2_V_2_O_7_ (PDF#73-1032) and monoclinic Cu_3_V_2_O_8_ (PDF#74-1503), respectively. Moreover, the intimate contact of Cu_2_V_2_O_7_ and Cu_3_V_2_O_8_ noticeably appeared in Figure 2d, demonstrating the formation of Cu_2_V_2_O_7_/Cu_3_V_2_O_8_ composite. Based on the above XRD, FTIR, TEM and HRTEM results, it was concluded that CVC heterojunctions were successfully constructed [54].

**Figure 3:**
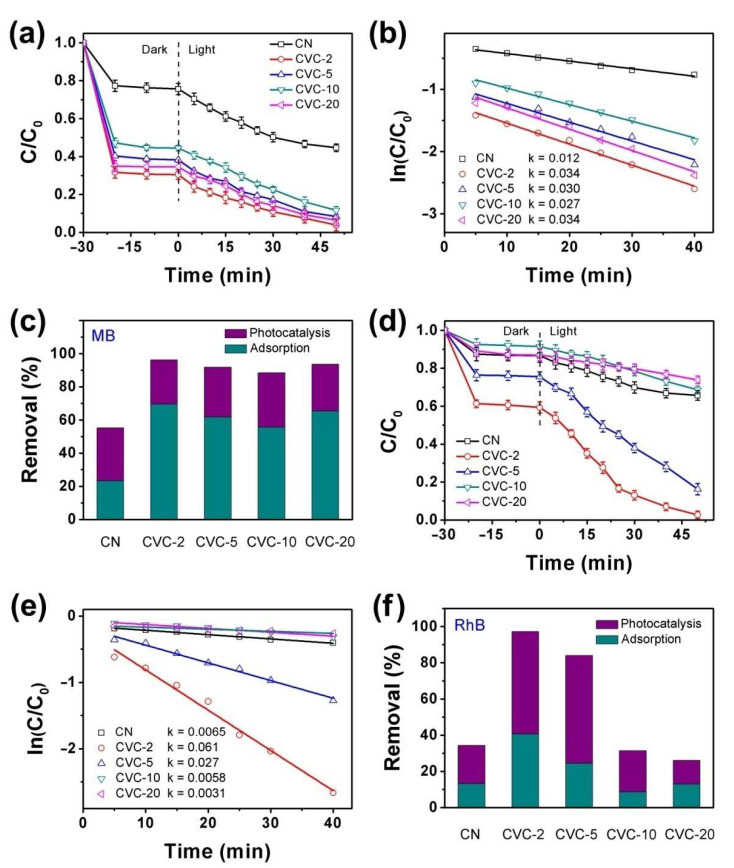
Adsorption and photocatalysis synergistic removal of (**a**) MB (concentration: 5 mg/L) and (**d**) RhB (concentration: 5 mg/L) over CN and CVCs (dosage: 100 mg) under 40 W white LED irradiation, fitted by pseudo first order rate equation and the rate constants (k) for photocatalytic degradation of (**b**) MB and (**e**) RhB, the total adsorption and photocatalysis degradation efficiency of (**c**) MB and (**f**) RhB.

The adsorption and photocatalysis performance of dyes (MB and RhB) over CN and CVCs were investigated and the results are depicted in Figure 3. As shown in Figure 3a,d, the adsorption–desorption equilibrium was achieved within 30 min for all samples. The adsorption capacities of MB and RhB on CVC-2 were 3 times that of CN. The enhanced adsorption capacity of CVC-2 indicated the stronger interaction between CVCs and dyes. All CVCs had much higher adsorption capacity of MB than CN. There was no necessary relation between the adsorption capacity and CV content, suggesting that the changed surface charge that resulted from the addition of CV nanoparticles was not the main factor to affect the interaction between CVCs and dyes. The larger specific surface area profited from the thermal etching effect in the reheating synthesis process which improved the adsorption capacity. The adsorption and photocatalytic degradation results revealed in Figure 3a,d manifest that CVC-2 exhibited the best overall performance for removal of dyes, although it removed MB mainly by adsorption and eliminated RhB primarily via photocatalysis (Figure 3c,f). The kinetic data of MB and RhB photocatalytic degradation on CN and CVCs were well fitted by a pseudo first order rate equation (Figure 3b,e). The degradation rate constants of MB and RhB on CVC-2 were, respectively, 0.036 min^−1^ and 0.061 min^−1^, which were about 3 times and 10 times that of pristine CN. The total removal efficiency of MB and RhB on CVC-2 was 96.2% and 97.3%, respectively. It was much higher than that of CN (55.3% for MB and 34.3%for RhB). This could be considered as the result of the synergistic effect of adsorption and photocatalysis.

**Figure 4:**
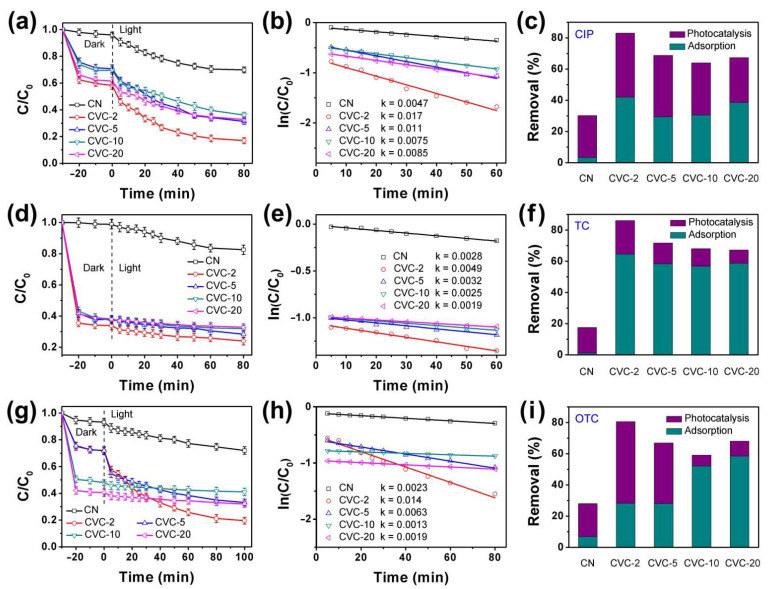
Adsorption and photocatalysis synergistic removal of (**a**) CIP (concentration: 4 mg/L), (**d**) TC (concentration: 40 mg/L) and (**g**) OTC (concentration: 20 mg/L) over CN and CVCs (dosage: 100 mg) under 40 W white LED irradiation, fitted by pseudo first order rate equation and the rate constants (k) for photocatalytic degradation of (**b**) CIP, (**e**) TC and (**h**) OTC, the total adsorption and photocatalysis degradation efficiency of (**c**) CIP, (**f**) TC and (**i**) OTC.

Antibiotics (CIP, TC and OTC) were also selected as the target contaminants to further investigate the synergistic removal effect of adsorption and photocatalysis on CVCs. The results are displayed in Figure 4. As presented in Figure 4a,d,g, the adsorption–desorption equilibrium was achieved within 30 min for all samples. The adsorption capacity of CVCs was much higher than that of pristine CN. The larger specific surface area improved the adsorption capacity. CVC-2 exhibited the best overall performance for removal of antibiotics, although it removed TC mainly by adsorption and eliminated CIP and OTC primarily via photocatalysis (Figure 4c,f,i). The adsorption capacity of CVC-2 was 16.9 (CIP), 58.6 (TC) and 4.2 times (OTC) that of CN, respectively. The total removal efficiency of CIP, TC and OTC on pristine CN was 30.2%, 17.5% and 28.0% during the adsorption and photocatalytic degradation process. In contrast, it was 83.0%, 86.0% and 80.5% for CVC-2, respectively. This was considered as the result of the synergistic effect of adsorption and photocatalysis. All CVCs exhibited considerably higher total removal efficiency than CN, indicating the excellent adsorption and photocatalysis performance of CVCs. From Figure 4c,f,i, we found that CVC-2 possessed highest photocatalytic activity. By comparison, the photocatalytic activity of CVC-5, CVC-10 and CVC-20 was decreased with the increase in CV content. This might be derived from the reaction active sites on the surface of CN nanosheets that were excessively occupied by CV nanoparticles in CVC-5, CVC-10 and CVC-20 [16,32]. Moreover, the removal efficiency of dyes and antibiotics on CVC-2 was compared with various recent reported results. As depicted in Table S1, the diversity degradation activities were higher than literature values [25,28,52,55,56,57,58,59], signifying that CVC-2 is probably a valuable catalyst of practical application in environmental sewage treatment. The kinetic data of CIP, TC and OTC photocatalytic degradation on CN and CVCs were well fitted by a pseudo first order rate equation (Figure 4b,e,h). The degradation rate constants of CVC-2 were 0.017 min^−1^ (CIP), 0.0049 min^−1^ (TC) and 0.014 min^−1^ (OTC). These were 3.6, 1.8 and 6.1 times that of pristine CN, respectively. This result indicated that the loaded CV nanoparticles on the surface of CN nanosheets could improve the photocatalytic activity.

**Figure 5:**
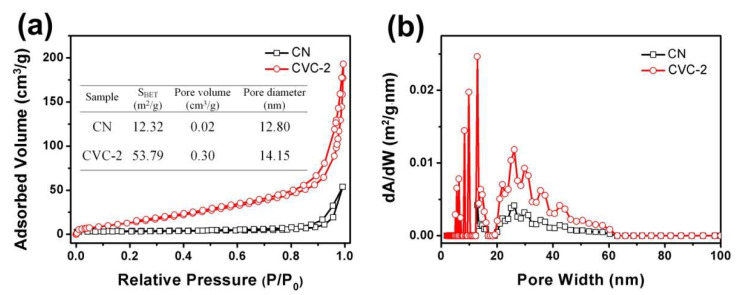
(**a**) the N_2_ adsorption–desorption isotherms and (**b**) the corresponding BJH pore size distribution curves of CN and CVC-2.

The BET surface area, pore volume and average pore diameter of CN and CVC-2 were investigated by N_2_ adsorption–desorption isotherms. As displayed in Figure 5a, the adsorption–desorption isotherms possessed the features of type IV curves, suggesting the samples had a mesoporous structure [60]. The H3 hysteresis loop at high P/P_0_ manifested that the mesopores of CN and CVC-2 were irregular. The BET surface area, pore volume and average pore diameter of CVC-2 were evidently greater than those of CN (Figure 5a, inset). They were, respectively, around 4.4, 15.0 and 1.1 times those of CN, which might result from the thermal etching in the reheating synthesis process [61]. The BJH pore size distribution of CN and CVC-2 revealed the wide pore size distribution from 20 to 60 nm, which probably resulted from the aggregation of CN in the reheating synthesis process as displayed previously in the SEM results (Figure S1a). The pore size between 10 and 20 nm originated from the porous structure of CN, which was observed previously in TEM images (Figure 2a,b). Therefore, the increased specific surface area, pore volume and average pore diameter could be instrumental in providing more adsorption and photocatalytic reaction active sites, and thus finally improve the synergistic effect of adsorption and photocatalysis.

**Figure 6:**
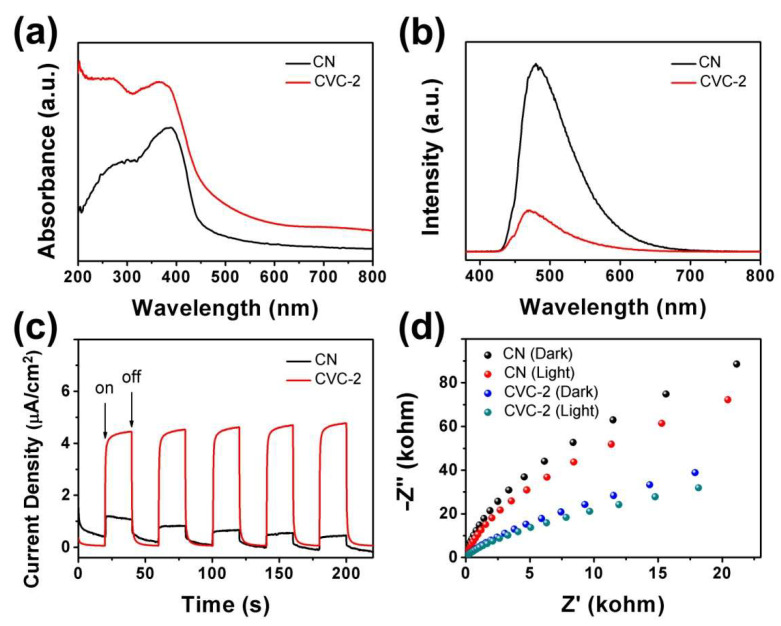
(**a**) UV–Vis diffuse reflectance spectra, (**b**) PL spectra, (**c**) TPC spectra and (**d**) EIS Nyquist plots of CN and CVC-2.

UV–Vis diffuse reflectance spectra of CN and CVC-2 are exhibited in Figure 6a. The absorption edge of pristine CN was at 457 nm, corresponding to a band gap of 2.71 eV. It was in agreement with the result reported previously [32,52], confirming that pristine CN had proper band gap for visible light harvesting. The absorption edge of CVC-2 was at 488 nm. The red shift of the absorption edge of CVC-2 suggested that the incorporation of CV nanoparticles onto CN nanosheets was instrumental in extending the visible light absorption range. From the DRS spectra in Figure 6a, it could be observed distinctly that CVC-2 possessed stronger light absorption in the wavelength range of 400–800 nm than pristine CN nanosheets. This could result in the improvement of the visible light-driven photocatalytic activity, which was clarified by the adsorption and photocatalysis degradation of CIP, TC and OTC depicted in Figure 4. The photoluminescence spectra (PL) were used to investigate the separation and recombination process of photogenerated charge carriers in CN and CVC-2 (Figure 6b). The higher PL intensity commonly indicates that photogenerated charge carriers have lower separation efficiency and faster recombination rate [32]. The PL emission peak intensity of CVC-2 was remarkably weaker than that of CN. It corroborated that the separation and recombination of photogenerated charge carriers was effectively ameliorated while CVC heterostructure was formed by incorporating CV nanoparticles into CN nanosheets. It could boost the photocatalytic activity of CVCs. The property of separation and recombination of the charge carriers in CN and CVC-2 could be further acquired from the photochemical measurements. TPC spectra and EIS Nyquist plots of CN and CVC-2 are exhibited in Figure 6c,d. The TPC spectra revealed that CVC-2 had better photostability and higher photocurrent density than CN (Figure 6c). The photocurrent density of CVC-2 was about 2.6 times that of pristine CN. It suggested that the photogenerated charge carriers possessed higher transfer rate in CVC-2, which was beneficial to the migration of the charge carriers to the surface of CVC-2 and consequently to improving its photocatalytic activity. EIS Nyquist plots of CN and CVC-2 are shown in Figure 6d. The arc radius of CN and CVC-2 under visible light irradiation was smaller than that detected in the dark. It demonstrated that the photoelectrode conductivity would be increased under the light irradiation condition. In addition, the arc radius of CVC-2 was less than that of pristine CN in either case, indicating that CVC-2 had higher photogenerated charge transfer efficiency. It was consistent with the previous PL and TPC results, demonstrating that the formation of CVC heterostructure could effectively facilitate the separation and transfer of the photogenerated charge carriers, suppress their recombination and finally improve the photocatalytic activity. The deduction obtained from the DRS, PL, TPC and EIS measurements conformed to the photocatalytic degradation experimental results of dyes and antibiotics.

**Figure 7:**
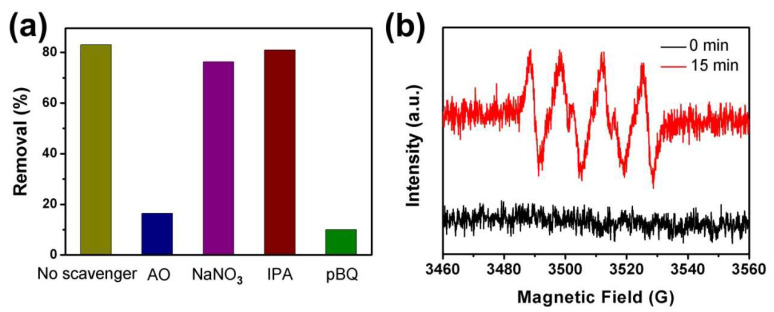
(**a**) The effect of radical scavengers on CIP removal efficiency over CVC-2 under 40 W LED irradiation and (**b**) ESR spectra of DMPO-•O_2_^−^ in methanol with CVC-2 in CIP removal system under different light irradiation times.

The reactive species involved in the photocatalytic degradation reaction were evaluated by radical scavenger experiments. NaNO_3_, ammonium oxalate(AO), isopropyl alcohol(IPA) and p-benzoquinone (pBQ) were adopted as the scavengers to trap e^−^, h^+^, •OH and •O_2_^−^ in the photocatalytic degradation of CIP over CVC-2, respectively. As revealed in Figure 7a, the removal efficiency of CIP was reduced dramatically after adding AO and pBQ, suggesting that h+ and •O_2_^−^ were the major reactive species in the photocatalytic process. Comparatively, just a slight reduction of degradation efficiency was observed with the addition of NaNO_3_ and IPA. It indicated that e^–^ and •OH hardly participated in the photocatalytic reaction. To further confirm the production of •O_2_^−^, ESR spectra of DMPO-•O_2_^−^ over CVC-2 in the CIP photocatalytic system were detected under different light irradiation times and the result is presented in Figure 7b. No obvious ESR signals of •O_2_^−^ were found in the dark, but they appeared after 15 min of visible light irradiation. These results demonstrated that •O_2_^−^ was generated under the visible light irradiation and participated in the photocatalytic degradation process of CIP.

**Figure 8:**
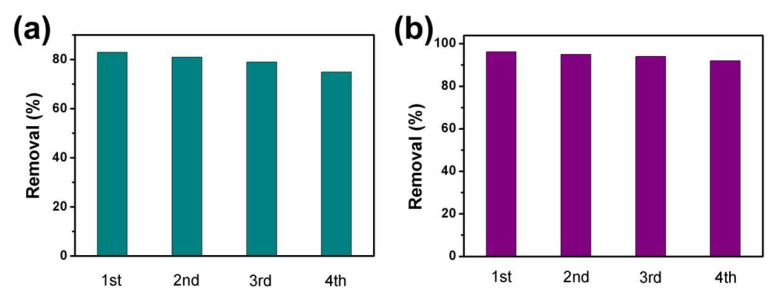
The stability experiments for the synergistic removal of (**a**) CIP and (**b**) MB over CVC-2.

The stability was assessed by four cycles of the adsorption and photocatalysis synergistic removal experiments of MB and CIP on CVC-2. CVC-2 was centrifuged and desorbed in deionized water several times to eliminate absorbed MB and CIP after each cycle experiment. As shown in Figure 8a,b, the removal efficiency of MB and CIP was well maintained after four degradation runs, confirming that CVC-2 had superior stability and excellent potential application prospects in wastewater treatment. TEM, XRD and FTIR were used to characterize the differences of structure and morphology of CVC-2 before and after the synergistic removal experiment. As shown by the TEM, XRD and FTIR results depicted in Figures S3–S5, the phase structure and morphology characteristics were not changed evidently after four degradation runs, which further illustrated the excellent stability of CVC-2.

The energy band structure of CN and CV was obtained by DRS and XPS VB spectra. The DRS spectrum of CV is displayed in Figure S6. It exhibited the absorption edge of 628 nm, corresponding to the band gap of 1.97 eV. It was in agreement with thatβ-Cu_2_V_2_O_7_in the literature [62]. XPS VB spectra of CN and CV are shown in Figure S7. The E_VB_ of CN and CV was 1.58 eV and 2.20 eV, respectively. The E_CB_ of CN and CV could be estimated based on the following equation: E_g_ = E_VB_ − E_CB_. It was -1.13 eV and 0.23 eV, respectively. The E_CB_ of CV was more positive than the redox potential of O_2_/•O_2_^−^ (−0.33 V) [52], meaning that O_2_ adsorbed on CV could not be reduced to •O_2_^−^. Therefore, the reduction reaction of O_2_ could only occur on the CB of CN. ESR spectra of DMPO-•OH were also measured under visible light irradiation with the presence of CVC-2 (Figure S8). They confirmed the production of •OH although the radical scavenger experiments indicated that •OH radicals did not participate in the photocatalytic reaction. Furthermore, the E_VB_ of CN was more negative than the redox potential of •OH/OH^−^ (+1.99 V) and •OH/H_2_O (+2.37 V) [63]. The •OH radicals could not be generated on CN, and only formed on the VB of CV. These results clearly suggested that CVC heterojunctions possessed the characteristics of Z-scheme photocatalytic systems.

**Figure 9:**
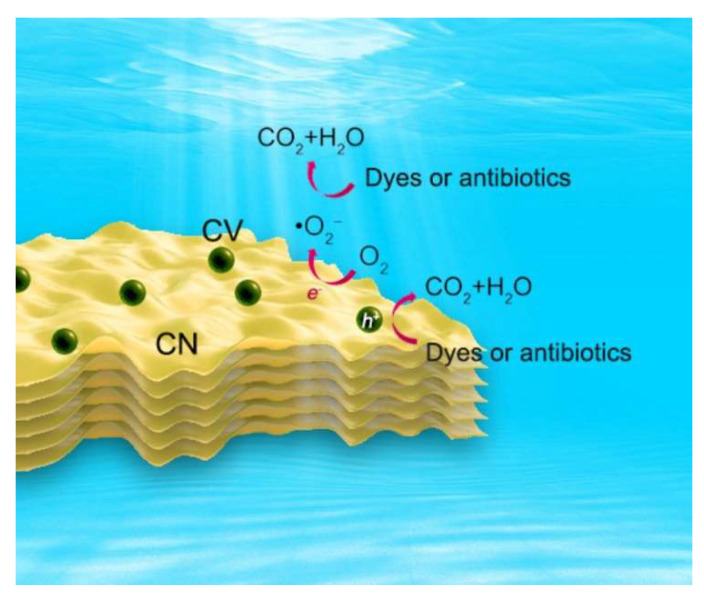
The possible mechanism for photocatalytic degradation of dyes and antibiotics on CVCs.

The possible photocatalytic mechanism of dyes and antibiotics on CVCs is consequently proposed in Figure 9. Firstly, the photoinduced electrons (e^−^) were excited and transferred to the CB of CN and CV under visible light irradiation. The photoinduced holes (h^+^) were still retained in the VB. Secondly, the electrons in the CB of CV transferred to CN and recombined with the holes located in the VB of CN. This recombination could significantly accelerate the separation of the photoinduced electrons and holes of CN and CV (Equation(1)), which was the advantage of Z-scheme heterojunctions [64,65]. Thirdly, the electrons on CN reduced oxygen to generate •O_2_^−^ and then •O_2_^−^ further oxidized dyes and antibiotics (Equations(2) and (3)). The holes on CV directly oxidized dyes and antibiotics to small molecules (Equation(4)). The photocatalytic degradation process of dyes and antibiotics could be elaborated as follows:

CVC-2 + hν→ CN (e^−^) + CV (h^+^), (1)

CN (e^−^) + O_2_→ •O_2_^−^, (2)

Dyes and antibiotics + •O_2_^−^→ degraded products, (3)

Dyes and antibiotics + CV (h^+^) → degraded products. (4)

## 3. Materials and Methods

### 3.1. Synthesis of CVCs

Preparation of CN nanosheets: 1:1 mole ratio of dicyandiamine and NH_4_Cl was mixed and calcinated for 4 h at 550 °C [61]. One hundred micrograms of obtained powder was ground and dispersed in 100 mL deionized water. The mixture was treated ultrasonically for 8 h and then centrifuged for 10 min at 5000 rpm. The as-prepared CN nanosheets in the supernatant were separated by vacuum freeze-drying.

Preparation of CVCs: 100 mg of CN nanosheets, 1:1 mole ratio of Cu(NO_3_)_3_ and NH_4_VO_3_ was dispersed in 100 mL of deionized water. Then, the water was evaporated and annealed at 500 °C for 2 h. A series of CVCs were synthesized by adjusting the mass ratio of CV and CN. The as-prepared CVC-2, CVC-5, CVC-10 and CVC-20 contained 2, 5, 10 and 20 wt% of CV, respectively. CV nanoparticles were synthesized under the same conditions except for the absence of CN nanosheets.

### 3.2. Catalyst Characterization

X-ray diffraction (XRD) patterns of CN, CV and CVCs were tested on a Rigaku Smartlab diffractometer equipped with a Cu-Kα radiation source. Transmission electron microscopy (TEM) and high-resolution TEM (HRTEM) images of CVCs were taken on a JEOL-2100F system. Fourier-transform infrared spectra (FTIR) of CN and CVCs were detected on a Nicolet NEXUS 470 spectrometer in the range of 4000–500 cm^−1^. Scanning electron microscopy (SEM) images, energy dispersion spectrum (EDS) and elemental mapping images of CVC-2 were characterized on a JSM-4800F scanning electron microscope. X-ray photoelectron spectroscopy (XPS) spectra of CN, CV and CVCs were obtained on a Thermo ESCALAB 250XI spectrometer equipped with an AlKα X-ray source. The BET specific surface area and N_2_ adsorption desorption isotherms of CN and CVCs were recorded on a Micromeritics ASAP 2460 analyzer at 77 K. The ultraviolet–visible diffuse reflectance spectra (DRS) of CN and CVCs were taken on a Shimadzu UV-2401 spectrophotometer equipped with an integrating sphere accessory.

### 3.3. Adsorption and Photocatalytic Experiments

The adsorption of contaminants on CN, CV and CVCs was implemented in the dark before the photocatalysis process. Typically, 50 mg CN, CV or CVCs was dispersed in 100 mL antibiotic (CIP, TC and OTC) or dye (MB and RhB) solution. The pH value of the solution was adjusted using 0.1 M HCl or NaOH. The mixture was stirred continuously in the dark for 30 min to reach adsorption–desorption equilibrium of the contaminants on the catalysts. The photocatalytic degradation of contaminants was evaluated under the visible light irradiation of 40 W white LED. Five milliliters of solution was fetched out at intervals and the catalyst was removed through a 0.22 μm PTFE filter membrane. The contaminant concentration in adsorption solution was detected by a UV–Vis spectrophotometer at 277 nm (CIP), 357 nm (TC), 352 nm (OTC), 664 nm (MB) and 552 nm (RhB). The temperature was fixed at 25 °C in the adsorption and photocatalysis process. The tests were repeated three times.

The stability of CVC-2 was appraised by 4 cycles of adsorption photocatalytic degradation experiments. CVC-2 was centrifuged and desorbed in deionized water several times after each cycle experiment to eliminate absorbed contaminant. Then, CVC-2 was separated and freeze-dried for the usage in the next cycle experiment. The reproducibility of CVC-2 was evaluated by repeating the above process twice. NaNO_3_, AO, IPA and pBQ were selected as the scavengers to trap e^−^, h^+^, •OH and •O_2_^−^, respectively.

### 3.4. Photoelectrochemical Measurement

The photoelectrochemical measurements were conducted on the CHI 660E electrochemical workstation. Ag/AgCl was the reference electrode and Pt foil was the counter electrode. A 0.2 M Na_2_SO_4_ solution was the electrolyte. For the preparation of working electrode, 5 mg of CN or CVCs was mixed with 0.2 mL Nafion and 1.8 mL ethanol ultrasonically. Then, the mixture was dropped on 1 cm^2^ of FTO glass and dried naturally. The transient photocurrent response (TPC) was tested under the irradiation of 40 W white LED. The irradiation intervals were realized using a mechanical light chopper. The electrochemical impedance spectroscopy (EIS) was measured at a frequency from 100 to 0.01 Hz.

## 4. Conclusions

In summary, CVC heterojunctions with ultrathin structure were successfully prepared by the reheating synthesis process for the adsorption and photocatalysis synergistic removal of various dyes and antibiotics. The thermal etching process increased the specific surface area of CVCs. The formation of heterojunctions enhanced the visible light absorption and improved the separation efficiency of photoinduced charge carriers. These factors simultaneously ameliorated the adsorption capacity and photocatalytic degradation performance of CVCs. CVC-2 exhibited the best synergistic removal efficiency of MB (96.2%), RhB (97.3%), CIP (83.0%), TC (86.0%) and OTC (80.5%). These photocatalytic degradation processes followed the pseudo first order equation. The pseudo first order rate constants of MB, RhB, CIP, TC and OTC photocatalytic degradation on CVC-2 were 3, 10, 3.6, 1.8 and 6.1 times those of pristine CN, respectively. DRS, XPS VB and ESR results suggested that CVCs had the characteristics of Z-scheme photocatalytic systems. Moreover, superoxide radicals and photoinduced holes were proved to be the major active species in the photocatalytic degradation process. This work provides a reliable reference for environmental sewage treatment by the adsorption and photocatalysis synergistic process.

## Data Availability

The original data are available from the corresponding author upon reasonable request.

## Supplementary Materials

The following supporting information can be downloaded at: https://www.mdpi.com/article/10.3390/ijms232214264/s1.
