# Supplementary material for: The Synergistic Effect of Adsorption-Photocatalysis for Removal of Organic Pollutants on Mesoporous Cu2V2O7/Cu3V2O8/g-C3N4 Heterojunction
Source: Int J Mol Sci. 2022 Nov 17;23(22):14264. doi: 10.3390/ijms232214264 (PMC9693244; doi:10.3390/ijms232214264)
Supplement: Supplementary file 1 [file ijms-23-14264-s001.zip › ijms-2017703-supplementary.pdf]

The synergistic effect of adsorption-photocatalysis for removal of organic pollutants on mesoporous $Cu_2V_2O_7/Cu_3V_2O_8/g\text{-}C_3N_4$ heterojunction

Jian Feng, Xia Ran, Li Wang, Bo Xiao, Li Lei, Jinming Zhu, Zuoji Liu, Xiaolan Xi, Guangwei Feng, Zeqin Dai*, Rong Li*

Engineering Research Center for Molecular Medicine, School of Basic Medical Sciences/School of pharmacy, Guizhou Medical University, Guiyang 550025, China

* Corresponding author. Tel.: +86 851 88174017
E-mail address: lirong1@gmc.edu.cn (Rong Li); daizeqin300@163.com (Zeqin Dai)

Table S1. Comparison of adsorption and photocatalysis removal efficiency of dyes and antibiotics on CVC-2 with various catalysts

Photocatalysts	Reaction Conditions	Removal efficiency %	Reference
CVC-2	80 min, 40 W white LED	MB (96.2%), RhB (97.3%), CIP (83.0%), TC (86.0%), OTC (80.5%)	this work
MCN/rGOA	80 min, 300 W Xe-lamp	RhB (95.2%)	[25]
AC(5%)/g-C_3N_4	120 min, 300 W Xe-lamp	RhB (97%)	[28]
g-C_3N_4-ZnO@GA(30%)	120 min, 300 W Xe-lamp	RhB (82.7%)	[52]
Ni_2P/BMO/CN	120 min, 500 W Xe-lamp	OTC (38.5%), TC (80.1%), CIP (36.0%)	[55]
g-C_3N_4-CdS	180 min, 500 W Xe-lamp	90.45% (MB)	[56]
hm-CN	120 min, 300 W Xe-lamp	RhB (92.73%), MB (56.95%), TC (73.7%), CIP (24.08%)	[57]
$CuBi_2O_4/Bi_2MoO_6$	60 min, 300 W Xe-lamp	TC (72.8%), OTC (74.0%), CIP (36.7%)	[58]
$Ag_2O/Bi_{12}GeO_{20}$	120 min, 250 W Xe-lamp	TC (65%)	[59]

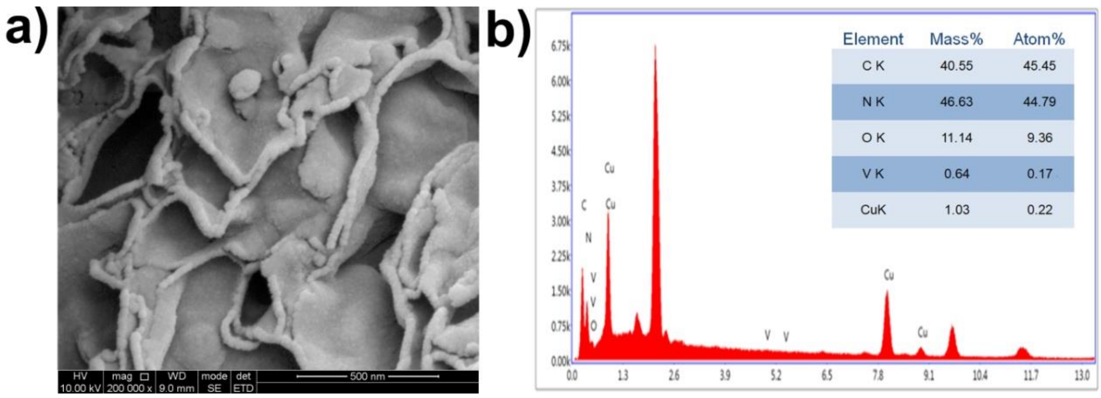

Figure S1 a) SEM image and b) EDS spectrum of CVC-2

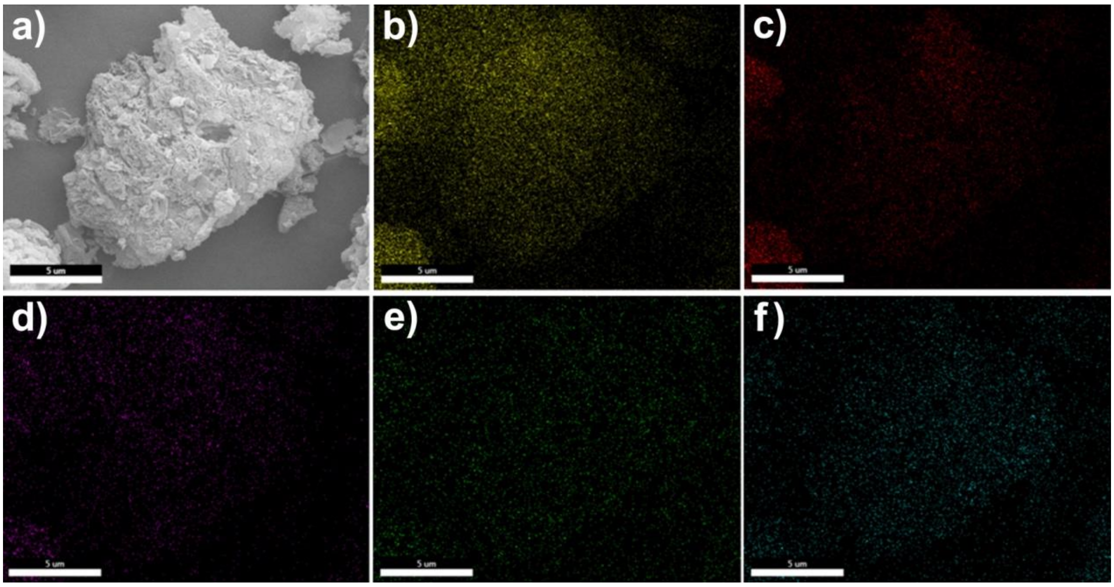

Figure S2 SEM image and elemental mapping images of CVC-2. a) SEM image and element mapping of b) C, c) N, d) O, e) Cu and f) V. Scale bar = 5 μm.

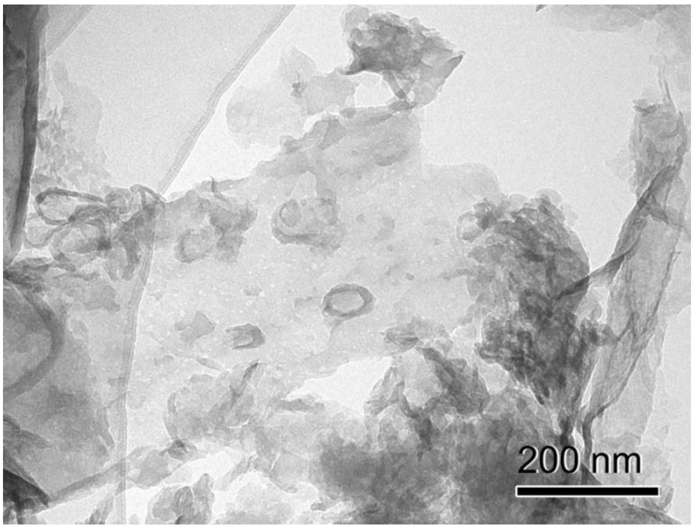

Figure S3 TEM image of used CVC-2

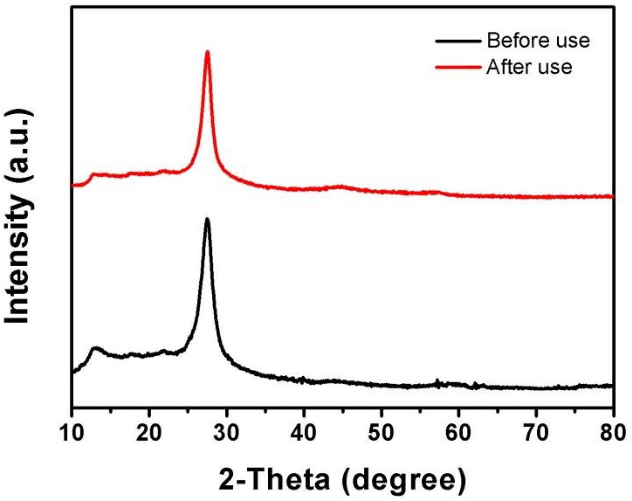

Figure S4 XRD patterns of CVC-2 before and after used

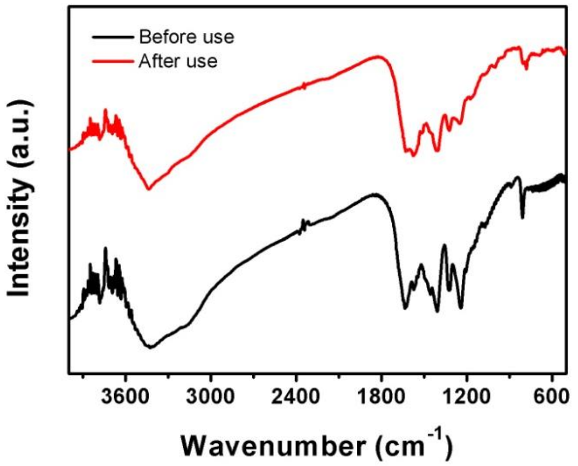

Figure S5 FTIR spectra of CVC-2 before and after used

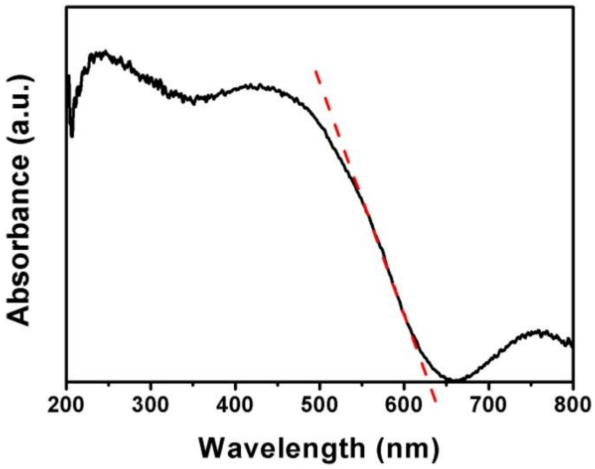

Figure S6 UV-vis diffuse reflectance spectra of CV

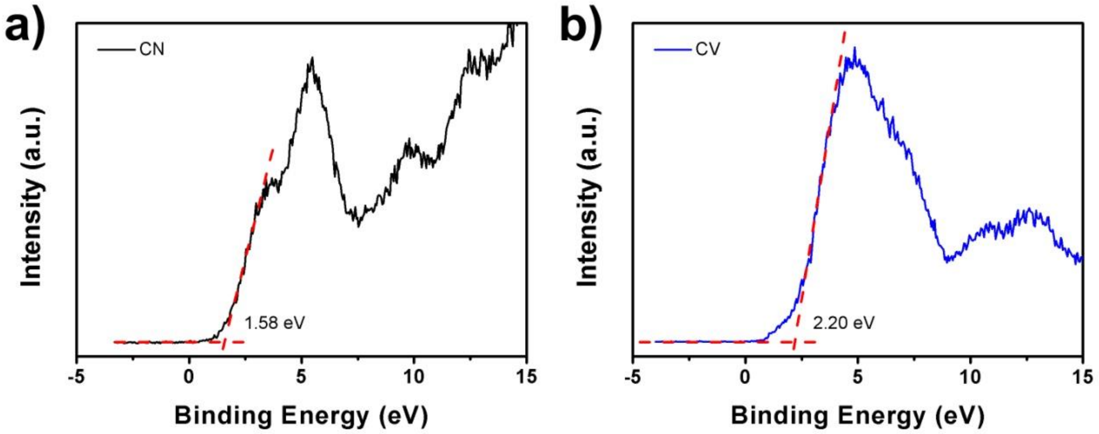

Figure S7 XPS VB spectra of CN and CV

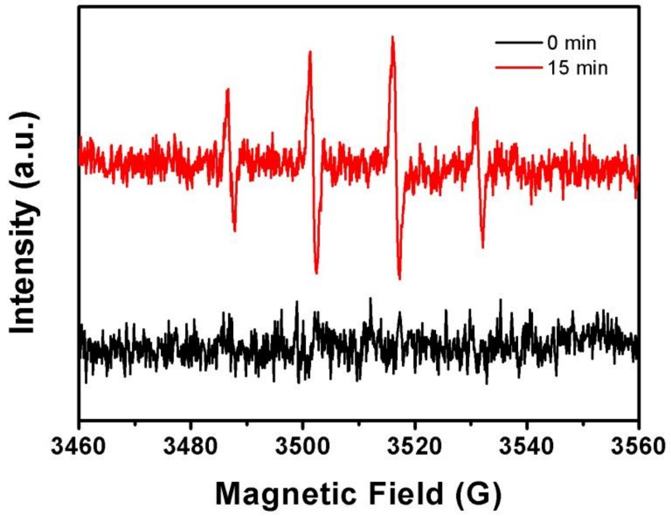

Figure S8 ESR spectra of DMPO-·OH in water with CVC-2 under different light irradiation time
